# Application of Genomic Big Data in Plant Breeding: Past, Present, and Future

**DOI:** 10.3390/plants9111454

**Published:** 2020-10-28

**Authors:** Kyung Do Kim, Yuna Kang, Changsoo Kim

**Affiliations:** 1 Department of Bioscience and Bioinformatics, Myongji University, Yongin 17058, Korea; kyungdokim@mju.ac.kr; 2 Department of Crop Science, Chungnam National University, Daejeon 34134, Korea; dkwl3120@cnu.ac.kr; 3 Department of Smart Agriculture Systems, Chungnam National University, Daejeon 34134, Korea

**Keywords:** next-generation sequencing, single nucleotide polymorphism, marker-trait association, genomic selection, marker-assisted selection

## Abstract

Plant breeding has a long history of developing new varieties that have ensured the food security of the human population. During this long journey together with humanity, plant breeders have successfully integrated the latest innovations in science and technologies to accelerate the increase in crop production and quality. For the past two decades, since the completion of human genome sequencing, genomic tools and sequencing technologies have advanced remarkably, and adopting these innovations has enabled us to cost down and/or speed up the plant breeding process. Currently, with the growing mass of genomic data and digitalized biological data, interdisciplinary approaches using new technologies could lead to a new paradigm of plant breeding. In this review, we summarize the overall history and advances of plant breeding, which have been aided by plant genomic research. We highlight the key advances in the field of plant genomics that have impacted plant breeding over the past decades and introduce the current status of innovative approaches such as genomic selection, which could overcome limitations of conventional breeding and enhance the rate of genetic gain.

## 1. Introduction

The history of plant breeding might have begun when humans changed from hunter gatherers to farmers, otherwise known as an agricultural society. The key concept of early plant breeding was “crop domestication”, which implies the adaptation of wild plant species, so that humans could sustainably cultivate food plants to feed an ever-increasing population. Researchers estimate crop domestication dates back 9000 to 11,000 years. Since then, humans have bred plants using selection schemes according to the decisions of farmers. In addition, humans have historically traded crop plants from other countries for hundreds of years, which has diversified the agricultural environment and facilitated plant breeding [1].

**Figure 1:**
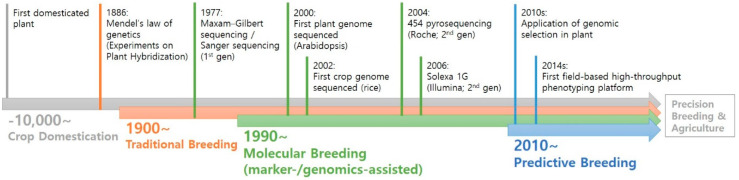
Key technological milestones in plant breeding.

The concept of scientific breeding started in the 19th century, triggered by Gregor Mendel’s research with garden peas, for which his plant hybridization led to his laws of inheritance. At the time of publishing his research paper, “Experiments on Plant Hybridization”, his work failed to get the attention of other scientists because biology was always interpreted by quantitative matters not by qualitative approaches [2]. However, his work was re-discovered by William Bateson in 1900. After integration with the Boveri–Sutton chromosome theory, scientists finally conceded his experiment was “the core of classical genetics”. Consequently, plant breeding faced a new era by the advances of modern genetics with a plethora of newly generated breeding populations in many crops. In other words, the full-scale application of transmission genetics to plant breeding had begun at that point (e.g., introgression of useful traits in other accessions). However, at the beginning, plant breeding was only performed by phenotype evaluations, meaning that a breeder’s ability to select desirable individuals was very critical. Soon after this, the breeding environment dramatically changed ever since the Maxam–Gilbert sequencing method was published in the late 1970s. The method was the first to provide nucleotide sequence information that could directly be used for developing molecular markers, which are very objective and have a capability for early stage selection. After the application of this method in plant genetics, first generation (Sanger sequencing), second generation (next-generation sequencing technology mostly referring to Illumina chemistry), and third generation sequencing technologies (Pacific BioScience or Oxford Nanopore technologies) have emerged and had significant impacts on plant breeding in a variety of ways [3]. For example, researchers started to utilize nucleotide information by applying molecular marker systems such as single nucleotide polymorphisms (SNPs), which are theoretically infinite in terms of numbers in plant genomes, enabling expanded marker-assisted selection (MAS) or genomic selection (GS) [4]. Additionally, having the whole genome sequence information is very powerful in plant breeding because the locations of traits and their underlying genes or chromosomes could be helpful for the introgression of interesting traits to other accessions. In addition, transcriptome sequencing (RNAseq) has revealed gene expression changes under various abiotic or biotic stress conditions, leading to profiles of genes associated with many agricultural traits (Figure 1).

In lectures on “Plant Genetics” at some universities, professors always define genomics as “the study of genetics with massive nucleotide sequence information”. In fact, plant breeding based on genetic tools is to identify genetic variants of interest that can be associated with phenotypic differences. In this respect, genomics provides breeders and geneticists with new opportunities in that modern molecular genetics have offered a relatively limited number of genetic variations whereas whole genome information can theoretically provide an unlimited number of genetic variations based on SNPs. Thus, the power of the whole genome reference becomes a critical tool for this purpose. As stated, sequencing technologies advance quickly, and the cost is decreasing. The massive amount of genetic variants thanks to technological advances has enabled breeders to find marker-trait associations (MTA), which exploit the genotyping of various populations with molecular markers covering the whole genome and analyze the associations between phenotypic variations and genotypic polymorphisms. Thus, researchers may be able to expand their application of conventional MAS techniques to the use of big data, which results in genomics-assisted selection (GAS). The expanded selection scheme has caused more precise dissection of genomes, which may improve breeding programs by reducing linkage drags, false positive markers, etc. [5]. In addition, we now face a new paradigm of plant breeding by predicting segregating models of specific traits based on a training population with the genotype and phenotype data. The established model (expressed as genomics-evaluated breeding values, GEBVs) is used to select target plants in the breeding populations after testing those in a validation population. The concept of this GS is originally based on old statistics and bioinformatics that use training sets and validation sets, but it is also based on one of the top-notch techniques, machine learning (ML) [6].

In this review, the impact of genomics on modern plant breeding efforts is discussed, covering from sequencing technologies and whole genome references to their applications in breeding such as high-throughput genotyping and GS. We hope this review helps researchers by summarizing the overall history and advances of plant breeding together with the advances in plant genomics.

## 2. Application of Genomic Tools to Crop Improvement

### 2.1. History of Nucleotide Sequencing Technologies

Research on the structure of DNA has been conducted by scientists for a long time, but relatively recently, research has been conducted to obtain the genetic information contained in DNA. The development of nucleotide sequencing has greatly contributed to the ability to easily obtain a lot of genetic information. The history of nucleotide sequencing technology can be divided into three major generations [7].

The so-called first-generation sequencing refers to Maxam–Gilbert sequencing and Sanger sequencing. The Maxam–Gilbert method is the principle of decomposing bases using a chemical reaction using formic acid, dimethyl sulfate, or hydrazine on the 5′-end of a DNA fragment with ^32^P-labeled, and then confirming it by polyacrylamide gel electrophoresis [8]. The principle of Sanger sequencing is a chain-termination technology developed by Frederick Sanger in 1997. Dideoxynucleotides (ddNTPs) that end the DNA chain during DNA replication are inserted into the DNA polymerase in a limited way. As ddNTPs lack the 3′-OH required to form a phosphate ester bond, the DNA polymerase stops extending the DNA. Using this principle, the method is to add each of the four dideoxynucleotides (ddATP, ddGTP, ddCTP, ddTTP), combined with fluorescent substances, to DNA and polymerize them with polymerase, and then check the base sequence using gel electrophoresis or chromatography [9]. Sanger sequencing was used to read genome sequences from plants such as rice and *Arabidopsis thaliana*, but it was still time-consuming and expensive [10].

Since the mid-2000s, the development of next generation sequencing (NGS) technology has enabled the production of large quantities of sequence data quickly, cheaply, and accurately. First, the 454 technology was developed in 2008. Its principle concept is to make emulsion oil using hydrophilic and hydrophobic properties and then to amplify by emulsion PCR by fixing one DNA strand to one bead. In the amplification process, the two phosphates that occur when a base is combined are turned into ATP, resulting in chemical luminescence as oxyluciferin is generated, which is the signal that identifies the base sequence. This method can read long sequences, but when a homopolymer is produced, the exact sequence cannot be determined, and the sequence is produced in a smaller amount than other second-generation sequencing techniques [11]. Second, it is most commonly used in second-generation technology as sequencing by synthesis (SBS) technology. SBS technology connects a short adapter oligo to DNA fragments and amplifies it in a flow cell containing a complementary oligo to form a cluster. When forming a cluster, dNTPs with fluorescent substances that emit different wave lengths of light depending on the type of base are synthesized one by one by DNA polymerase. At this time, the principle is to read and record the wave length of the fluorescent material, which is combined at each base, and repeat this process about 100 times to read the sequencing [12]. Using this method, it is possible to obtain a large amount of data quickly, accurately, and at a low cost, but it has the disadvantage of mainly obtaining a short sequence. Third, the method of amplification used in ion torrent uses emulsion PCR, such as in the 454 series, but the method of detection uses the semiconductor sequencing method. The method of electrically measuring hydrogen ions is based on the fact that hydrogen ions are generated each time a base is added. However, it is not electrically distinguishable because any additional base produces the same hydrogen ion. Therefore, hydrogen ions are measured after reacting with dATP [13]. Second-generation sequencing has the advantage of sequencing a single DNA molecule into a single base, so it is possible to sequence many DNA fragments simultaneously in a small amount, but amplification is necessary for signal detection. However, third-generation sequencing can read very long DNA sequences by sequencing single DNA without amplification of DNA molecules.

The third-generation sequencing technology refers to PacBio’s single molecule real time (SMRT) technology (www.pacb.com) and Oxford Nanopore technology (ONT) (www.nanoporetech.com). SMRT sequencing is a technology called zero-mode waveguide (ZMW), which performs sequencing by recognizing the fluorescence of complementary binding bases as DNA chains pass through the DNA polymerase fixed at the bottom of a small hole. This method has the advantage of being able to produce a long read of more than 20Kb [14]. The ONT sequencing performs sequencing by forming a nanometer-level channel in a membrane and then passing through a single strand of DNA, changing the potential difference between the membranes according to the base sequence. From the outside of the membrane, the double-stranded DNA is released by a helicase enzyme, and the single strand is configured to move into the channel at a constant rate. Unlike other methods, this method does not undergo any other complementary chemical reaction to the template DNA, and the DNA sequence can be read as it is. In addition, it has the advantage that sequencing is possible with a personal computer or a laptop using a cartridge that can be connected through a USB port, not a special device [15].

### 2.2. The Whole Genome References

The decreasing cost of nucleotide sequencing has changed the landscape of building the whole genome reference of many plant species. Nonetheless, the sequenced genomes have various different levels in terms of completeness. For example, some references have reached the level of pseudomolecules, which have been anchored to the chromosome scale, while many references have been published as a draft status. For plant breeders, any references with pseudomolecules will be really powerful because all the loci information can be acquired for the traits of interest, leading to the precise development of molecular markers for large-scale MAS and the easy detection of candidate genes. Draft genomes can also contribute to crop breeding thanks to their help in accurate genotyping using nucleotide sequence information.

As of October 2020, a total of 699 genomes in Viridiplantae have been sequenced as many different forms. The details (names of species, forms of assemblies) are well presented in the NCBI’s genome database (www.ncbi.nlm.nih.gov/genome/browse#!/overview/viridiplantae) and the database is updated whenever a new genome assembly is added. The information is actively used for a variety of plant sciences. Additionally, other databases, such as Plant GDB (www.plantgdb.org) or Phytozome (www.phytozome.org), are providing plant genome information. Table 1 shows the formats and versions of genomics data for representative crop species deposited in those two databases. Once, assembling plant genomes was very challenging because of some specific features such as being relatively large and having highly repetitive genomes. In addition, plant genomes have abundant polyploidy or have experienced paleopolyploidization, resulting in complicated genomic structures due to paralogous or homologous sequences. Consequently, whole genome plant references in the early 21st century spent a vast amount of resources using first-generation sequencing technology (see previous section for details). However, the quality of the reference genomes was somewhat guaranteed in terms of their completeness. Some model species such as *Arabidopsis* [16], rice [17], maize [18], sorghum [19], populous [20], grapevine [21], papaya [22], or soybean [23] were sequenced at that time. After that, NGS technologies covering the second and third generation sequencing methods dramatically reduced the costs and resolved some assembling issues associated with plant genome structures. As a result, over 250 angiosperm species have been completely sequenced so far, and the number keeps rapidly increasing with or without constructing complete pseudomolecules [24]. Global statistics of sequenced genomes are well-documented in the Genome Database for Angiosperms (GDA, www.angiosperms.org) with appropriate web links. The point is that whole genome resources have facilitated the development of plant breeding programs as these resources enable researchers to provide precise genotyping schemes, which are directly usable for breeders in their field trials in the form of genomics-assisted breeding (GAB) based on the expansion of the MAS or GAS concepts. We do not discuss the details of the whole genome references in this review because there are a plethora of reviews regarding this subject [24].

### 2.3. High-Throughput Genotyping and the Necessity of Phenotyping

**Figure 2:**
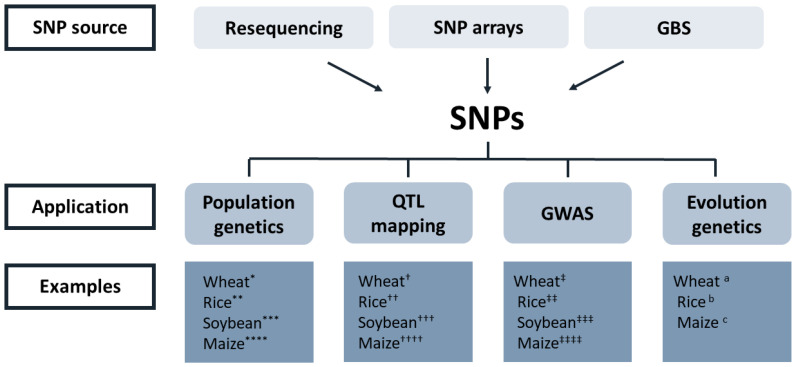
Applications of single nucleotide polymorphisms (SNPs) in plant sciences. Citations are as follows: * [47,48], ** [49], *** [50,51], **** [52,53,54], ^†^ [55,56,57], ^††^ [58,59,60], ^†††^ [50,61], ^††††^ [62,63,64], ^‡^ [65,66], ^‡‡^ [67,68,69], ^‡‡‡^ [70,71], ^‡‡‡‡^ [62,72], ^a^ [73,74,75], ^b^ [76,77], ^c^ [78,79].

**Figure 3:**
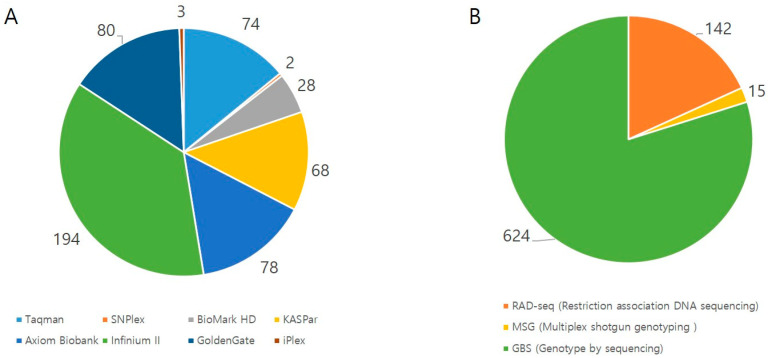
Publication search results (the NCBI’s PubMed database, www.pubmed.gov) from 2011 to present using different SNP genotyping methods in plant sciences. (**A**). Array- or PCR-based genotyping platforms. (**B**). Next generation sequencing- (NGS)-based genotyping platforms.

Genotyping is a major process for plant breeding in that accurate plant breeding requires a number of plant individuals with a certain level of genetic variation. At the beginning of Mendelian genetics, genotyping mostly relied on their phenotypic variations, limiting target traits for plant breeding. The advent of the polymerase chain reaction (PCR) method has drastically advanced genotyping technology as a superior tool using PCR-based molecular markers such as random amplification length polymorphism (RAPD) and amplified fragment length polymorphism (AFLP). These marker types were popular choices for many studies because they do not require nucleotide sequence information and are cheap; however, the marker information is not reproducible in different populations. Ever since first-generation sequencing became commercially available, sequence-based PCR markers have become predominant in genotyping procedures. For example, simple sequence repeat (SSR) markers are relatively inexpensive, abundant in plant genomes and more informative than previous PCR-based markers [40]. The most powerful aspect of SSRs were synergized together with the development of expressed sequence tags (ESTs), which capture actively expressed genes. EST-SSRs were once scientists’ favorite choice because they can link marker information and genes associated with target traits. Nonetheless, we cannot define SSR as a tool for high-throughput genotyping because gel-based genotyping SSRs are very laborious and time-consuming, and automated fragment analysis systems are relatively generally low-throughput with high cost even if they provide a certain level of multiplexing. The most recent molecular marker is single nucleotide polymorphisms (SNPs), which are theoretically unlimited in plant genomes. The reason why scientists chose SSRs over SNPs from the 1990s to early 2000s was that SNP discovery and genotyping with DNA sequencing was extremely expensive and complicated. However, NGS technologies have made SNPs the primary choice for many breeding studies due to their high flexibility, speed, and cost-effectiveness [41,42]. SNP markers have the potential to be universally used for genotyping from different sources, enabling integrated analysis across different species due to certain levels of similarities in nucleotide sequences. Although there are some ambiguous interpretations in some polyploidy species because of their biallelic nature, SNPs are still the most popular in modern genotyping experiments. The examples of SNP applications are shown in Figure 2 with some citations. As a consequence, a variety of concepts and methods have been adopted and used for SNP genotyping pipelines. The high-throughput application of SNPs for plant breeding can be largely divided into array- or PCR-based SNP genotyping platforms and NGS-based sequencing genotyping platforms. When the number of samples are small, the cost-effectiveness of these high-throughput genotyping methods may not meet our expectation but if one researcher has a fairly good number of samples, array- or PCR-based genotyping platforms can drastically reduce the cost per data point by virtue of their high levels of multiplexing. Of course, an NGS-based method is also high-throughput because it uses an NGS system to find SNPs based on the depth of the sequence information. While array- or PCR-based genotyping platforms require a priori knowledge of the nucleotide sequence information, the NGS-based method does not need it. Therefore, NGS-based genotyping can be widely used for species that do not have a reference genome, but it is not as accurate as array- or PCR-based platforms and not reproducible for different trials. The details of those platforms were previously discussed [41]. We think that the popularity of those platforms for the last decade needs to be checked in the current review article. To date, array- or PCR-based SNP genotyping platforms such as Taqman (Applied Biosystems), SNPlex (Applied Biosystems), BioMark HD (Fluidigm), KASPar (LGC), Axiom Biobank (Affymetrix), Infinium II (Illumina), GoldenGate (Illumina), and iPlex (Sequenome) are commercially available. Some of those are being actively used in plant sciences based on the number of publications in the NCBI’s PubMed database (www.pubmed.gov). Thanks to the flexibility of NGS platforms, a variety of NGS-based pipelines have been applied to plant sciences such as restriction association DNA sequencing (RAD-seq) [43], multiplex shotgun genotyping (MSG) [44] and genotype-by-sequencing (GBS) [45]. Poland and Rife summarized the NGS-based genotyping methods in their review article [46]. Herein, we integrate NGS-based genotyping to GBS because GBS seems the most popular method according to the publication search in the NCBI’s PubMed database. Figure 3 shows searchable publications using various genotyping platforms applied to plant sciences from the NCBI’s PubMed database from 2011 to present.

For array- or PCR-based platforms, Taqman (multiplexed only) has been widely used for plant sciences. Axiom, GoldenGate, KASPar, and Infinium II have been moderately similar in terms of their use in searchable publications (Figure 3A). In fact, researchers may use the same platforms once they build an array of information, so the tendency may not dramatically change for the next few years.

The whole genome reference is a desirable gadget for calling SNP variants; however, GBS is relatively cost-effective because it generates partial genomic sequences utilizing restriction digestion. Although the reduced cost of nucleotide sequencing has popularized GBS for SNP analysis, genotyping errors, or missing data due to low coverage may be the biggest obstacle for SNP genotyping. In particular, as stated, it is a bit difficult to apply this technique for polyploidy crops, which can complicate SNP calling because of paralogous or homologous sequences. Nonetheless, for those species with no reference information, GBS may be the only option for SNP genotyping and discovery. As researchers already recognize that whole genome deep sequencing is not necessary for only genotyping purpose, reduced representation library (RRL) sequencing has been applied to the SNP genotyping procedure using NGS technology. Largely, three different methods are being used to date as stated, but GBS seems the most popular choice for plant science (Figure 3B). Those three methods are basically the same concept with restriction digestion and the ligation of bar-coded adaptor sequences. Thus, many modified procedures have been published such as double-digested RAD-seq [80], double-digested GBS [81], Ion Torrent GBS [82], and restriction fragment sequencing (REST-seq) [83]. Thus, researchers can choose adequate methods that may be the best fit for their experimental designs. Considering the advance in analysis techniques, genotyping methods would not be an issue for plant breeding.

High-throughput phenotyping (HTP) is also an indispensable methodology and needs to be briefly mentioned, in order to coordinate genomics data with breeding programs. Once, phenotyping required time-consuming and laborious processes. In other words, the number of phenotypic data points could be a stumbling block for combining phenotypic variations with genotypic variants. However, a variety of high-throughput phenotyping platforms (HTPP) have been introduced to plant breeding in the past few years thanks to the advancement of pre-existing technologies such as novel sensors, image analysis, robotics, and remote-sensing data mining (previously reviewed in Araus and Cairns [84]). HTTP enables plant breeders to evaluate numerous agricultural traits in a fast and accurate manner, which could be matched with the huge amount of genotypic data.

### 2.4. Genomics-Assisted Selection and Breeding

The whole genome reference is obviously omnipotent for crop breeding and genetics research because it enables gene discovery, positioning the gene location, accurate marker assignment, and the development of high resolution maps [85]. However, time, effort, and costs may be the biggest stumbling block for building pseudomolecular levels. Researchers already recognize that the whole genome reference is somewhat overpaid material for accomplishing a specific purpose. In other words, researchers may have to think about cost-effectiveness in order to not waste their resources to achieve their default targets. Plant breeders evaluate a variety of genetic materials such as core collections, bi-parental populations, diversity panels, breeding populations, and mutant lines. Here, we discuss some genomic tools that may be best applied for various breeding materials.

#### 2.4.1. Characterization of Genetic Resources Using Genomics Tools

Plant breeding initially requires genetic variants bearing elite alleles for target traits. Breeders generally use a variety of genetic resources to obtain their desirable alleles such as well-designed bi- or multi-parental populations, diversity panels, core collections, and even intermediate breeding lines. The previously stated genotyping platforms can accelerate connecting genotype information to phenotypes, which is comparable to conventional methods in terms of an experimental size. The capacity to take care of a large number of individuals has enabled breeders to more precisely dissect genomic parts of interest based on the high resolution of the linkage disequilibrium (LD) based on a detailed haplotype analysis. A new concept was created, genomics-assisted breeding (GAB), thanks to their expanded and precise marker-assisted selection, leading to a new revolution in plant breeding, particularly for complex traits. The selection of pre-stated genotyping platform is, therefore, very important for cost-effective breeding programs. All the genetic populations listed above can make use of different SNP genotyping platforms, but there may be some things to consider. We do not discuss a resequencing method in this section due to its cost-effective issue despite its superior strength and accuracy in crop genomics. Commercial SNP-genotyping arrays are available for some major crops (refer to the Table 1 by You et al.) [86], which may be the most accurate and cost-effective way for high-throughput genotyping. If there is a reference genome for a certain crop but no commercial SNP array is available, in-house SNP arrays or reference-based GBS may be good options; however, in order to build an in-house SNP array, a prior knowledge of sequence information is necessary by genome resequencing; therefore, GBS may be the best choice in this case. GBS is relatively accurate and reliable when the whole genome reference is available; however, for those species without any reference genomes, GBS will be the only option for genotyping although the results will not be as accurate as other approaches. The point is that a variety of genotyping platforms enable plant breeders to observe more genomic breakpoints so that the introgression of target traits or genes into the elite cultivars has less deleterious effects, such as linkage drags.

When breeders cannot find desirable alleles, sometimes mutant lines (artificially- or naturally-induced) are investigated. For facilitating the identification of mutant alleles, genomic tools known as target induced local lesions in genomes (TILLING) [87] or ecotype TILLING (EcoTILLING) [88] are useful. These methods use some specialized nucleases, *CEL*I or *Endo*I, which recognize and cut mismatched DNA sequences. In terms of excavating useful variants, the methods may be suitable for linking mutant genotypes to phenotypes without sequencing the whole genome. These methods have been applied to some major crops such as rice [89], wheat [90], barley [91], and maize [92].

#### 2.4.2. Marker-Trait Associations for Expanded Marker-Assisted Selection

Conventional plant breeding was dependent upon a breeder’s ability to make phenotypic evaluations, meaning that the result of elite selection could be biased by the breeders, and therefore, a bit subjective. The problem arises particularly for complex traits using phenotype-based selection because complex phenotypes show continuous variation in a breeding population, indicating that a number of breeding accessions must be grown in the field in order to capture desirable variations, which are limited by labor, time, costs, space, environment, and so on. Ever since the PCR technique became predominant in plant science from 1980s, the initial concept of MAS was formed by adopting molecular markers in plant breeding programs with their objectivity and reproducibility, thanks to the denser dissection of genomes. Consequently, capturing genetic variants in various populations associated with agriculturally important traits underlying candidate genes has become a critical field in crop genetics and breeding science. In fact, researchers have worked on an accurate MTA for a successful and efficient MAS.

At the beginning of the era of molecular markers, some tried single-marker analysis (SMA) for MTA. This method consisted of fitting a regression model and running an F test analysis of variance (ANOVA) at each marker locus with phenotypic data based on the appropriate statistical models [93]. In fact, this type of analysis provided the basis for modern association mapping methods. However, the SMA is optimized for common or rare alleles with a minor allele frequency (MAF) greater than 0.01; consequently, it has a very low power for rare alleles and sometimes is inflated by type I errors (false positives) [94]. Nowadays, the SMA is not frequently used for MAS but for a quick screening of genotype and phenotype associations. Two recent main streams for MTA using genomic tools are quantitative trait loci (QTL) analysis and genome-wide association study (GWAS). The detection of QTL is basically performed by linkage mapping with bi-parental populations. Newly applied genomics tools enhance the accuracy of this procedure by enabling increases in the number of markers mapped, securing high-density genetic maps. Although QTL can detect MTA linked to rare alleles because it can be artificially introduced, the disadvantage of QTL mapping is mostly caused by the limited number of crossovers due to short generations of mapping populations. Therefore, QTL mapping gives a low resolution while it provides a high statistical power for detecting major alleles associated with traits having high heritability. To avoid these limitations, the mapping resolution can be drastically improved by adopting newly designed bi-parental populations such as a multi-parent advanced generation intercross (MAGIC) population [95]. High-throughput genotyping partly resolves these advantages by enabling researchers to investigate a large number of mapping individuals. In contrast, GWAS can accommodate many more recombination events that occurred in the history of the analyzing population, improving the resolution of MTA. Diversity panel, core collections, and even nested-association mapping (NAM) populations can be analyzed with this new powerful tool by virtue of high-throughput genotyping. Despite the higher resolution of GWAS compared to QTL mapping, it can generate false positives or true negatives due to subpopulation structures of the analyzing populations, and it requires a relatively larger population size than QTL mapping to capture accurate genotypic and phenotypic variations. Consequently, researchers need to be cautious to select mapping individuals that have large genotypic variations and to detect accurate associations that can be used for downstream usages such as breeding or genetic research. Although a large collection of the population is used for GWAS, the entire process can still be compromised due to an increased genetic heterogeneity if the careful selection of population individuals is not considered [96]. Moderate to high levels of heterozygosity can cause inaccurate genotyping, particularly when using low-coverage genomic sequence data such as GBS. For species with a high level of heterozygosity, researchers need to secure more sequence coverage to avoid any genotyping errors. Polyploidy (or paleopolyploidy) can result in genotyping errors through the confusion between subgenomic (or paralogous) and allelic variations. As stated, there is no resolution for this issue due to the biallelic nature of SNPs unless reference genomes are well established. Another issue for GWAS is that rare alleles are overlooked during filtering genotyping data based on minor allele frequency (MAF). In most of the cases, genotype data are filtered at MAF ≥ 0.05, meaning that rare alleles can mostly be ignored. Unfortunately, rare alleles represent natural variations in some populations, which could be very important for breeding and genetic research. As stated, QTL may be more useful for rare alleles because bi-parental populations can be precisely designed by artificially introducing traits potentially associated with rare variants.

The final step for genomics-assisted breeding is marker-assisted selection of target accessions. Conventionally, selection was performed by breeders’ personal choice based on phenotypic differences. Modern selection schemes, however, mostly depend on molecular markers, which are very objective and abundant in plant genomes, together with the advance of MTA. Since molecular markers have emerged, MAS has been applied to breeding many crops such as rice [97,98], wheat [99,100], sorghum [101,102] and soybean [103,104]. A plethora of genomic data dramatically improved the efficiency of MAS by densely dissecting genomic regions associated with important agricultural traits. MAS combined with genomics has emerged as a crucial tool for plant breeding; however, it is still difficult to select some traits phenotypically, especially for complex traits affected by environmental conditions or developmental stages. Additionally, MAS does not provide solutions for some issues caused by linkage drags. The issue can be resolved if many breeding accessions (or mapping individuals) are grown and investigated, but it is practically impossible. For example, breeders cannot grow over thousands of breeding accessions for each research purpose because the seed-producing ability of crops is not infinite. Even if a breeder can grow many accessions, it is still impossible to dissect genomic regions if favor alleles are tightly linked to deleterious alleles. As an alternative, geneticists started looking at haplotypes based on linkage disequilibrium (LD). Genomics big data combined with haplotype data enable plant scientists to exploit all the genotype and phenotype data as predictors of the genomic estimated breeding values (GEBVs). The new concept created a new selection scheme called GS, which will be further discussed in the next section.

## 3. Predictive Genomics and Breeding

### 3.1. Genomic Selection (GS)

Artificial selection of plant individuals or populations with desirable traits is one of the most important processes of plant breeding. An accurate selection would enable plant breeders to maintain a smaller size of breeding population as well as shorten the time needed for developing a new variety. In the early history of plant breeding, selections were made based only on the plant phenotypes, which are largely explained by their genotypes but also could vary in different environments, especially for quantitative traits. Nowadays, with the use of genomic tools, simple traits governed by one or a few genes can be effectively selected before evaluating their phenotypes [105,106].

Although the current MAS methods as described above are very effective for deploying a few major effect genes, the methods are shown to be inaccurate in predicting quantitative traits, mostly the agronomically important traits such as yield, seed weight, and quality [107,108]. This limitation, even with dense markers, is majorly due to the statistical approach, which is inadequate for polygenic traits—it only uses markers that are significantly correlated with a trait and fails to capture many loci of small effect [109,110]. Moreover, a biparental population, commonly used in QTL identification, is insufficient to represent allelic diversity and genetic background of the breeding population. This limits the translation of desirable QTLs directly to the breeding population with the equivalent genetic effect.

To overcome these limitations, Meuwissen et al. [111] proposed GS, which uses whole-genome prediction models for estimating genetic values. GS uses all available markers covering the whole genome to estimate breeding values (referred to as genomic estimated breeding values; GEBV) rather than testing its significance, while traditional MAS uses a predefined subset of significant markers with the rest treated as having zero effect [112,113]. By implementing GS with highly dense markers, which can be obtained by whole-genome sequencing and/or genotyping, both large and small QTL effects can be fully captured [109,110]. Moreover, unlike QTL mapping, there is no need to search for significant QTL-marker associations for each trait. QTL studies are still valuable for understanding the genetic architecture of quantitative traits; however, when considering a breeder’s perspective, GS can reduce effort involved in maintaining individual biparental populations and mandatory steps to reassess the effects of QTLs within the breeding population. Therefore, by adapting GS to plant breeding, the selection of elite genotypes can be achieved more efficiently and faster than with phenotypic selection or MAS approaches, and this can eventually shorten the breeding cycle of many crop species.

To perform GS, two sets of populations are required: a training population (TP) and a breeding (or selection) population (BP). GS builds a prediction model based on the genotypic and phenotypic data of individuals in the TP. The trained models are then applied to predict the breeding values of the genotyped individuals in BP without knowing their phenotypes. Breeders can subsequently select the individuals that are predicted to have superior phenotypes based on the GEBVs. During this selection process, the phenotype evaluations of any desirable traits are not necessary at all, only a genomic profile of the individuals, which can be cost-efficiently evaluated by various genotyping platforms, is required. Thereby, GS provides the ability to select the elite individuals without any phenotypic measurements, and breeders can benefit enormously from this. With GS, breeding becomes faster as the generation intervals between selection cycles become much shorter. Breeders do not need to wait for the population to grow until they can reach the development stages for phenotyping evaluation of an agronomic trait. Elite individuals can be selected even without planting the seeds, and this tremendously reduces the time and cost of the selection process. For example, major seed companies such as Monsanto incorporate GS effectively with their seed-chipping technologies and speed breeding methods to perform selections at least two or three times in a year by advancing the generations back and forth between open fields, greenhouses, and nurseries. Moreover, breeding becomes more intensive as more candidates can be evaluated per selection cycle [114,115]. Breeders can handle and screen a larger size of a breeding population, and even exotic germplasm and wild relatives can be tested to estimate the potential breeding values before introducing them to the breeding population. In this way, the performances of individuals with diverse genetic backgrounds can be assessed with a pre-trained model at a target environment that is either expected or predicted. A prediction model trained at different growing conditions could be able to tell which individuals or genomic profiles would perform best under the changing climate [116,117,118]. Therefore, GS has great potential to harness genetic gain in many aspects of breeding strategies.

The advantages of GS over traditional MAS have led to its application in plant and animal breeding, especially in livestock breeding [119,120,121]. In comparison with crop plants, livestock such as cattle, pigs, and chickens have a limited number of offspring that can be produced per generation and have a higher cost per progeny to raise and test their production index. Due to these distinct aspects, livestock sectors adopted GS more rapidly and widely, starting with dairy cattle in 2008 and later with beef cattle and chickens [122,123,124]. GS has been proven to have great potential to accelerate genetic gain in livestock breeding because the early selection using GS has doubled genetic gain when compared with the conventional progeny testing [125,126]. As shown in livestock breeding, the potential of GS is not questionable in general. However, the GS approaches from livestock breeding cannot be simply applied to complex plant breeding.

On the other hand, in the plant sectors, the potential of GS was explored in trees [127,128] and major crops such as wheat, maize, cassava, and soybean [50,116,129,130]. Although the true gain by GS remains unproven yet in plants, several results show that this approach is very effective, as seen in livestock; for example, proposed GS schemes for wheat breeding has estimated three to five times more genetic gain than that of the classical breeding program [114]. However, the implementation of GS to breeding programs has been limited to major international seed companies and public-funded programs for major cereal crops. This contrasting rate of GS application between plant and animal fields might be due to contrasting tools and resources available for breeders since plant breeders have several additional options in addition to GS to reduce generation times (e.g., double-haploid induction and speed breeding) and to replicate clones and inbreds (e.g., self or vegetative propagation) compared to animal breeders [131]. Nevertheless, to accelerate the wider application of GS in plant breeding programs, plant-specific approaches that differ from GS approaches in livestock breeding should be applied [132]. Recently, Xu et al. [133] suggested several requirements that should be considered for the extensive use of GS in plants. Although most of the suggestions are similar to those in livestock, the authors suggested that building a better prediction model with high-throughput, cost-effectiveness, and precision phenotyping is the most important consideration for plants. This is because very few plants are produced in a controlled environment while most of the industrialized livestock are produced in environment-controlled facilities. Phenotypic performances of plants are largely affected by genotype-by-environment interaction, which has been continuously emphasized in numerous pieces of literature (see Table 2 for examples) as the most important consideration to improve the prediction accuracy of GS in plants. [114,134,135,136].

Although improving phenomics and statistical models are the current obstacles to overcome for advancing predictive breeding, GS would still be not possible without cost-effective methods to profile the whole genome. These genotyping methods, either array-based or sequence-based, produce massive amounts of single nucleotide polymorphisms (SNPs), which are commonly used in GS for training models and calculating GBEVs. Instead of SNPs, however, a set of alleles referred to as haplotypes could give more accurate information for the expected phenotype. Previous studies have shown that haplotypes consisting of ten markers have yielded the highest estimation accuracy for breeding values [137,138]. Additionally, even with a low-coverage sequencing of individuals, unobserved genotypes can be imputed to reconstruct the target haplotype using machine learning and/or deep learning frameworks [139,140]. Therefore, haplotype-based models can be a cost-effective option for GS and have the potential to increase the accuracy of GS as well.

Moreover, assembling the haplotypes shared by the group of individuals showing superior performance of the target traits was proposed as a new approach for developing improved crops [141]. An assembled set of the superior haplotypes, which is referred to as a haplotype assembly, can be substantially selected for haplotype-assisted breeding [142]. Presumably, these superior haplotypes consist of beneficial alleles, which can be derived with the help of artificial intelligence (AI). As suggested in a recent review [143], deep learning models can be used to predict molecular phenotypes of given genotypes and further make ab initio predictions on unobserved sequence data. Neural networks (NNs) as well as deep learning-based natural language processing (NLP) models, such as bidirectional encoder representations from transformers (BERT) [144] and generative pre-trained transformer (GPT) [145] models, can be applied to predict superior haplotypes of the target traits without knowing the phenotypes.

**Table 2:** Examples of genomic prediction using genotype by environment (G × E) interaction models in wheat and maize.

Species	Size of Population	Number of Environments	Number of Genotyped Markers	Traits	References
Wheat	599	4	1279	Grain yield	[146,147,148,149]
Wheat	693	4	15,744	Grain yield	[149,150,151]
Wheat	670	4	15,744	Grain yield	[149,150,151]
Wheat	807	5	14,217	Grain yield	[149,150,151]
Wheat	557	5	12,083	Grain yield	[152]
Wheat	338	4	7594	Grain yield, days to heading grain volume weight, 1000-kernel weight	[153,154]
Wheat	287	18	~15,000	Grain yield, grain number, thousand-grain weight, thermal time for flowering	[155]
Wheat	297	3	1635	Fusarium head blight	[156,157]
Wheat	250	5	12,083	Plant height, days to heading	[151,158]
Wheat	767	4	2038	Grain yield, plant height, days to heading, days to maturity	[159,160]
Wheat	775	5	2038	Grain yield, plant height, days to heading, days to maturity	[159,160]
Wheat	964	4	2038	Grain yield, plant height, days to heading, days to maturity	[159,160]
Wheat	980	4	2038	Grain yield, plant height, days to heading, days to maturity	[159,160]
Wheat	329	4	7748	14 traits (including grain yield, plant height)	[161]
Wheat	8416 ^a^	3	39,758	Days to heading, days to maturity	[162]
Wheat	2374 ^a^	3	39,758	Days to heading, days to maturity	[151,158,162]
Maize	504	3	158,281	Grain yield	[116,148,149]
Maize	309	3	158,281	Grain yield, plant height, anthesis-silking interval	[116,151,158,163,164]
Maize	278	3	46,347	Gray leaf spot	[165,166]

^a^ landrace accessions.

### 3.2. Predictive Breeding and Agriculture

GS may be considered as the first step of predictive breeding and agriculture. The selection of predicted phenotypes by GS only comprises a small portion of the entire breeding process. A predictive and precisely measurable breeding practice can be applied to not only the selection step but also can be applied throughout the entire breeding process. For example, plant breeders can design an ideal crop plant under future climate conditions [167], and then, the prediction model may show a shortcut for developing a designed variety. The genome profiles of the entire breeding population and their interaction with various environments would suggest ideal decisions throughout the breeding program to reach the designed varieties [168]. These decisions were previously made based on breeders’ experience and intuition, which include the ideal combination of parental lines and the conditions for selection (or selective pressure).

**Figure 4:**
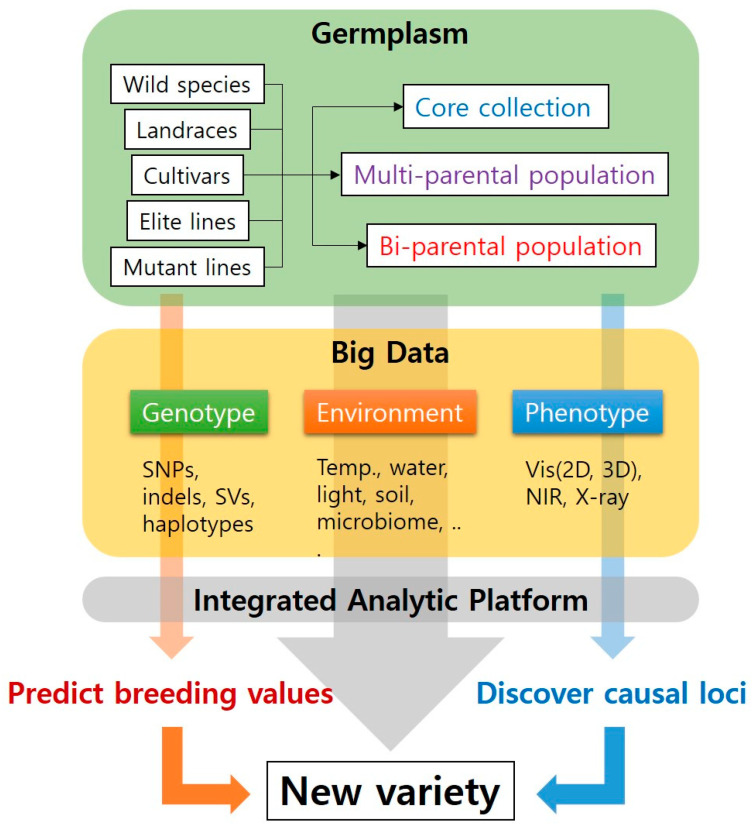
Flowchart of plant breeding in the era of genomics big data.

This improved decision-making during plant breeding could be expanded to agriculture. Massive advances and transdisciplinary efforts in genomics, phenomics, and artificial intelligence are guiding us into an era of predictive agriculture [169]. Accurate measurement and prediction methods for agriculture would provide appropriate genetic and management solutions at once (genotype by environment by management predictions) to prepare for the future environment of agriculture [168]. Breeders and agronomists will be able to identify the combination of which genetic features and agronomic practices are expected to maximize the potential of target crops. The integration of multi-omics data such as transcriptome, epigenome, and metabolome would help to increase the accuracy of the genotype by environment by management (GxExM) prediction [170,171]. For this improved genome-to-phenome prediction, big data ecosystems for plant breeding need to be built (Figure 4). Diverse germplasm and big data of genomes, phenomes, and environments together with integrative analysis fueled by AI will enable us to precisely identify casual loci and predict breeding values. This will eventually guide us to faster and smarter breeding decisions and management practices.

## 4. Future Directions

The tremendous advances in sequencing technologies and genomic tools have enabled us to explore vast amounts of genetic data from plant individuals in a cheaper and faster way. Plant breeders have so far successfully introduced these innovations and have developed numerous varieties with higher yield and better quality to ensure the food security of what we consume every day. In the coming decades, however, breeders will face more challenges to meet the global demands for food and feed. Several recent studies have repeatedly reported that climate change has a negative effect on global yields of many vital crops [172,173,174,175]. In addition to this, the global production of major crops needs to be increased by 60% in 2050 when considering the current trends in population and diets (e.g., 10 billion people with higher meat consumption) [176]. Even with the help of modern breeding techniques and platforms, the current rate of genetic gain is not enough to reach the projected food demands [177]. Plant breeders, therefore, need to find a more effective path to further enhance genetic gain and develop climate-change-ready varieties. As summarized in this review, many scientists and breeders suggest that genomic prediction and predictive breeding along with the big data of genomes and phenomes are the possible solutions to further increase the rate of genetic gain. Continuous production of genomic big data covering multi-omics data and an interpretation of the multi-dimensional data are also important to harness additional genetic gains. In the same context, private sectors have already incorporated these cutting-edge techniques into their breeding pipeline [178]. Moreover, interdisciplinary efforts could bring us big success as seen throughout the history of plant breeding, and genomics alone is not enough to innovate the current plant breeding paradigm. For instance, the advancements of HTP technologies would enable us to digitalize the detailed performances of plant individuals under different environments, and training these with phenome big data could increase the accuracy of the prediction model for GS [171]. Therefore, genomic prediction combined with technologies such as HTP, speed breeding, and/or genome editing could further boost the rate of genetic gain.

Even though genomic prediction is the most accurate method to select superior individuals, the approach requires profound resources of genomic and phenomic data and computational power. On the other hand, foreground selection with a single marker is still a very attractive method in most plant breeding programs. Therefore, different strategies and approaches should be applied separately for each breeding program after considering the available tools and resources. Most importantly, these applications need to be translated into delivering higher genetic gains in farmers’ fields, not just in breeders’ fields.

## Figures and Tables

**Table 1:** A representative species of genome served in a database, such as Plant GDB and Phytozome.

Species Name	Version	Data Base Type	Provider	References
*Arabidopsis thaliana* (Thale Cress)	AtGDB	Chromosome	Plant GDB	[25]
	TAIR10	Chromosome	Phytozome	
	Araport11	Chromosome	Phytozome	[26]
*Hordeum vulgare* (Barley)	HvGDB	BAC	Plant GDB	[27]
	r1	BAC	Phytozome	[28,29]
*Oryza sativa* (Rice)	OsGDB	Chromosome	Plant GDB	[30]
	v3.1 (Kitaake rice)	Chromosome	Phytozome	[31]
	v7_JGI	Chromosome	Phytozome	[32]
*Sorghum bicolor* (Sorghum)	SbGDB	Chromosome	Plant GDB	[33]
	Rio v2.1	Scaffold	Phytozome	[34]
	v3.1.1	Chromosome	Phytozome	[33]
*Solanum tuberosum* (Potato)	StGDB	Chromosome	Plant GDB	[35]
	v4.03	Chromosome	Phytozome	[36]
*Triticum aestivum* (Wheat)	TaGDB	BAC	Plant GDB	[37]
	v2.2	Chromosome	Phytozome	[38]
*Zea mays* (maize)	ZmGDB	Chromosome/BAC	Plant GDB	[39]
	Ensembl-18	EST	Phytozome	[18]
	PH207 v1.1	transcripts	Phytozome	[31]
